# Differentiation capacities of PS-clusters, adult pituitary stem/progenitor cell clusters located in the parenchymal-niche, of the rat anterior lobe

**DOI:** 10.1371/journal.pone.0196029

**Published:** 2018-04-23

**Authors:** Saishu Yoshida, Naoto Nishimura, Hideaki Yurino, Masaaki Kobayashi, Kotaro Horiguchi, Kentaro Yano, Shin-ichi Hashimoto, Takako Kato, Yukio Kato

**Affiliations:** 1 Division of Life Science, Graduate School of Agriculture, Meiji University, Kanagawa, Japan; 2 Organization for the Strategic Coordination of Research and Intellectual Property, Meiji University, Kanagawa, Japan; 3 Institute of Reproduction and Endocrinology, Meiji University, Kanagawa, Japan; 4 Graduate School of Medical Sciences, Kanazawa University, Kanazawa, Japan; 5 Department of Life Science, School of Agriculture, Meiji University, Kanagawa, Japan; 6 Laboratory of Anatomy and Cell Biology, Department of Health Sciences, Kyorin University, Tokyo, Japan; Lewis Katz School of Medicine at Temple University, UNITED STATES

## Abstract

Pituitary endocrine cells are supplied by *Sox2*-expressing stem/progenitor cells in the anterior lobe of the adult pituitary. In relation to their microenvironment (“niche”), SOX2-positive cells exist in two types of niches; the marginal cell layer-niche and the parenchymal-niche. Recently, we isolated dense stem/progenitor cell clusters from the parenchymal-niche as parenchymal stem/progenitor cell (PS)-clusters. We classified these PS-clusters into three subtypes based on differences in *S100β*-expression (S100β-positive, -negative, and -mixed type), and reported that S100β-positive PS-clusters exhibited the capacity for differentiation into endocrine cells under 3-dimensional cultivation system. In the present study, we further characterized S100β-positive PS-clusters using an *in vitro* 2-dimensional cultivation system. The results demonstrated that S100β-positive PS-clusters in the 2-dimensional cultivation system proliferated more actively than S100β-negative clusters. Moreover, in 2-dimensional cultivation conditions, S100β-positive PS-clusters showed differentiation capacity into non-endocrine cells (Myogenin-, αSMA-, NG2-, or SOX17-positive cells) but not into endocrine cells, whereas S100β-negative PS-clusters did not. Collectively, PS-clusters were heterogeneous, exhibiting different proliferation and differentiation properties based on the difference in *S100β*-expression. Specifically, a part of SOX2-positive cells in the parenchymal-niche had capacities for differentiation into non-endocrine cells, and S100β-positive PS-clusters may be in more progressive stages toward differentiation than S100β-negative clusters.

## Introduction

The anterior lobe of the pituitary gland is a key endocrine tissue containing five types of endocrine cells, i.e. growth hormone (GH)-, prolactin (PRL)-, thyroid-stimulating hormone (TSH)-, luteinizing hormone (LH)-, follicle-stimulating hormone (FSH)-, and adrenocorticotrophic hormone (ACTH)-producing cells. In addition to endocrine cells, a blood capillary network composed of endothelial cells, smooth muscle cells, and pericytes in the anterior lobe is necessary for maintaining the physiological functions of the pituitary. However, how these endocrine and non-endocrine cells are renewed during postnatal periods remains to be fully elucidated.

Recent studies by *in vitro* pituisphere forming assay [1] and *in vivo* cell tracing using *Sox2*^*CreERT2/+*^/*R26*^*YFP/+*^ mice [2] have demonstrated that *Sox2*-expressing stem/progenitor cells play a role in the physiological maintenance of the adult pituitary gland. Immunohistochemical analysis demonstrated that SOX2-positive stem/progenitor cells in the adult rat anterior pituitary are composed of sub-populations based on expression of several genes [3,4,5]. Among them, S100β-positive cells mainly start to appear on early postnatal day [6,7] and have been regarded as typical non-endocrine cells playing multi-functions as (1) phagocytes [8], (2) cells forming a cell-network via gap junctions [9], (3) supportive cells producing numerous growth factors [10–12], and (4) stem/progenitor cells [4,13]. *S100β*-positive cells in the adult rat anterior lobe are composed of 85% SOX2-positive cells [4]. Conversely, approximately 82% and 60% of SOX2-positive cells express *S100β* in the adult rat anterior lobe [4], and mouse anterior lobe [2], respectively.

In relation to the pituitary stem/progenitor cell microenvironment (or, “niche”) SOX2-positive cells exist in two types of niches; the marginal cell layer (MCL)-niche facing the residual lumen of Rathke’s pouch and the parenchymal-niche, composed of SOX2-positive cell clusters scattered in the parenchyma of the adult anterior lobe (parenchymal-niche) [14–16]. We recently isolated dense stem/progenitor cell clusters from the parenchymal-niche but not from the MCL, termed “parenchymal stem/progenitor cell (PS)-clusters” using the anterior lobe of S100β/green fluorescent protein-transgenic (S100β/GFP-TG) rats [17]. Among them, three subtypes of PS-clusters were identified based on S100β-GFP signals; i.e. GFP-, mixed-GFP-, and null-GFP-clusters, accounting for 47%, 37%, and 16% of PS-clusters, respectively. Notably, PS-clusters could not be dispersed by several enzymes such as 2.5% trypsin/5 mM EDTA [17]. Moreover, an *in vitro* differentiation assay demonstrated that approximately 18.8% of GFP-clusters differentiate into endocrine cells under 3 dimensional (3D)-cultivation in the presence of a GSK3β-inhibitor [17]. However, further characterization of their differentiation capacities especially into non-endocrine cells has not been performed.

It is known that cell plasticity and differentiation capacity differ depending on defined cultivation conditions such as 3D and 2D conditions [18]. Hence, in the present study, we attempted the further characterization of S100β-positive PS-clusters in 2D-cultivation conditions. Ultimately, S100β-positive PS-clusters showed unique capacities for differentiation into non-endocrine cells in the 2D-cultivation system.

## Materials and methods

### Ethics statement

All animal experiments were performed following approval from the Institutional Animal Experiment Committee of Meiji University (IACUC 14–0012) and were conducted in accordance with the Institutional Regulations of Animal Experiments and Fundamental Guidelines for Proper Conduct of Animal Experiments and Related Activities in Academic Research Institutions under the jurisdiction of the Japanese Ministry of Education, Culture, Sports, Science and Technology. All rats were sacrificed by cervical dislocation under anesthesia by diethyl ether, and did not become severely ill at any time prior to the experimental endpoint.

### Animals

Wistar-crlj S100β/GFP-TG rats generated by fusing the *S100β*-promoter to the reporter gene *Gfp* [19] were housed individually in a temperature-controlled room under 12 h light/darkness cycle conditions.

### Pituitary cell dispersion and isolation of PS-clusters

Cell dispersion of the anterior lobe of the rat pituitary and isolation of PS-clusters were performed according to a previous report [17]. Briefly, excised anterior lobes of the pituitaries from 2- to 5-month-old S100β/GFP-TG rats were treated with 0.2% collagenase (Sigma, St. Louis, MO USA) for 15 min at 37°C. After removal of the collagenase solution, collected cells were incubated in 10 mM HEPES-100 mM NaCl (pH 7.5; HEPES buffer) containing 0.25% trypsin (Sigma)-5 mM EDTA (Dojindo Laboratories, Kumamoto, Japan) for 10 min at 37°C. After removal of the trypsin solution by centrifugation, the collected cells were suspended in a mixed medium (Dulbecco’s modified Eagle’s medium (DMEM)/F-12) composed of DMEM and Ham F-12 (Life Technologies, Grand Island, NY, USA) without serum. The cell suspension was plated on a non-adhesive 35-mm dish (AGC Techno Glass, Shizuoka, Japan), and PS-clusters were immediately collected manually using pipettes under a microscope (Leica DM IRB, Leica, Wetzlar, Germany).

### Cell cultivation for differentiation

Collected PS-clusters were cultured on Matrigel-coated glass slides using growth factor-reduced Matrigel Basement Membrane Matrix (BD Biosciences, San Jose, CA, USA). Briefly, one or five manually collected PS-clusters were transferred into each well of 16-well chamber slides (Thermo Fisher Scientific) coated with growth factor-reduced Matrigel diluted 1:10 with DMEM/F-12 serum-free medium. To analyze the differentiation capacity of PS-clusters, the clusters were cultured for 7 days in three types of differentiation medium: 1) growth or differentiation medium (GD-medium) containing B27 supplement (1:50; Thermo Fisher Scientific), bovine serum albumin (BSA) (0.5%; Sigma), recombinant mouse basic fibroblast growth factor (bFGF) (20 ng/ml; R&D, Minneapolis, MN, USA), and recombinant human epidermal growth factor (EGF) (20 ng/ml; R&D) in DMEM/F-12, 2) activin-medium containing 100 ng/ml activin-A (kindly provided by Dr. Y. Hasegawa, Kitasato University, Japan) [20], N2 supplement (1:100; Wako, Osaka, Japan), and BSA (0.5%), or 3) a previously established differentiation medium reported [17] for 3D-cultivation containing bFGF (20 ng/ml; R&D), EGF (20 ng/ml; R&D), and 20% KnockOut Serum Replacement (KSR) (Thermo Fisher Scientific) for 4 days, followed by replacement with medium including 6-bromoindirubin-3′-oxime (BIO) (GSK3β-inhibitor, 250 nM; Wako) and cultivation for another 7 days. Cultivation was performed in a humidified atmosphere of 5% CO_2_ and 95% air. To evaluate the proliferative activity, PS-clusters were treated with bromodeoxyuridine (BrdU) (10 μM; Roche Diagnostics GmbH, Mannheim, Germany) in GD-medium at the time of 72 and 144 h, and immunostaining was performed at 24 h following BrdU-treatment. Cell viability testing of PS-clusters during cultivation was performed using ReadyProbes Cell Viability Imaging Kit (Thermo Fisher Scientific).

### Immunocytochemistry

For immunocytochemistry, PS-clusters prior to and following cultivation were fixed with 4% paraformaldehyde in 20 mM HEPES, pH 7.5, for 30 min at room temperature. For BrdU-staining, cells were treated with 1N HCl in HEPES buffer for 10 min at room temperature. Cells were incubated with blocking buffer containing 10% (v/v) fetal bovine serum and 0.4% (v/v) Triton X-100 in HEPES buffer for 60 min at room temperature. Primary antibody reaction was performed at an appropriate dilution (S1 Table) with blocking buffer overnight at room temperature. To detect pituitary hormone-positive cells, a cocktail of anti-pituitary hormone antibodies at an appropriate dilution (S1 Table) was used. After immunoreaction, cells were incubated with secondary antibodies using Cy3-, Cy5-, or FITC-conjugated AffiniPure donkey anti-goat, rabbit, mouse and guinea pig IgG, as well as chicken IgY (1:500; Jackson ImmunoResearch, West Grove, PA, USA). Cells were washed and incubated with VECTASHIELD Mounting Medium (Vector Laboratories, Burlingame, CA, USA) with 4, 6′-diamidino-2-phenylindole, dihydrochloride (DAPI). Immunofluorescence was observed under a DMI6000 B inverted microscope (Leica).

### Quantitative real-time polymerase chain reaction (qPCR)

Total RNA from non-cultured cells of rat anterior lobes and rat GFP-clusters cultured in GD-medium for 7 days were extracted using ISOGEN (Nippon Gene, Tokyo, Japan). Reverse transcripts were synthesized with PrimeScript Reverse Transcriptase (TaKaRa Bio, Otsu, Japan) using total RNA following DNase I treatment. Isolation of mRNA and synthesis of cDNA from non-cultured rat GFP- and null-GFP-clusters were performed as previously reported [21] with slight modification. qPCR was performed on an Applied Biosystems StepOnePlus Real-Time PCR system (Applied Biosystems, Foster City, CA, USA). Reactions were performed using SYBR Green Real-Time PCR Master Mix Plus (Toyobo, Osaka, Japan) and included 0.6 μM of a specific primer set for each gene (S2 Table). Each sample was measured in duplicate and data were quantified using the comparative C_T_ method (DC_T_ method) to estimate the gene copy number relative to TATA box binding protein (*Tbp*) as an internal standard [22]. Data are presented as the mean ± SE for three independent experiments. The DNA sequences of the PCR product from each sample, whose accession number are listed in S2 Table, were confirmed by nucleotide sequencing (data not shown).

### Statistical analysis

Immunocytochemical data regarding the proportions of BrdU-positive cells, SOX2-negative cells, Myogenin-positive cells, αSMA-positive cells, and NG2-positive cells were measured by counting 1000–6000 cells and 600–1,200 cells per experiment in cultured GFP-clusters and cultured null-GFP-clusters, respectively, and data are presented as the mean ± SE for three independent experiments. Data were validated using the *F*-test, and the statistical significance between the groups of GFP- and null-GFP clusters was determined by Student’s *t*-test. *P < 0.01.

QPCR data are presented as the mean ± SE (n = 3) in three independent experiments. Data were validated using One-way analysis of variance (ANOVA) followed by Tukey’s test for multiple comparisons performed using Prism Ver. 7 (GraphPad Software, San Diego, CA, USA). *P < 0.01.

## Results

### 2D-cultivation of GFP-clusters isolated from the anterior lobe of S100β/GFP-TG rats

Recently, we classified PS-clusters into three subtypes based on S100β-GFP signals and demonstrated that GFP-clusters can be differentiated into endocrine cells in the 3D-cultivation system [17]. Here, we examined the 2D-cultivation system using Matrigel-coated glass slides with respect to proliferation and differentiation capacities to further characterize GFP-clusters.

**Fig 1:**
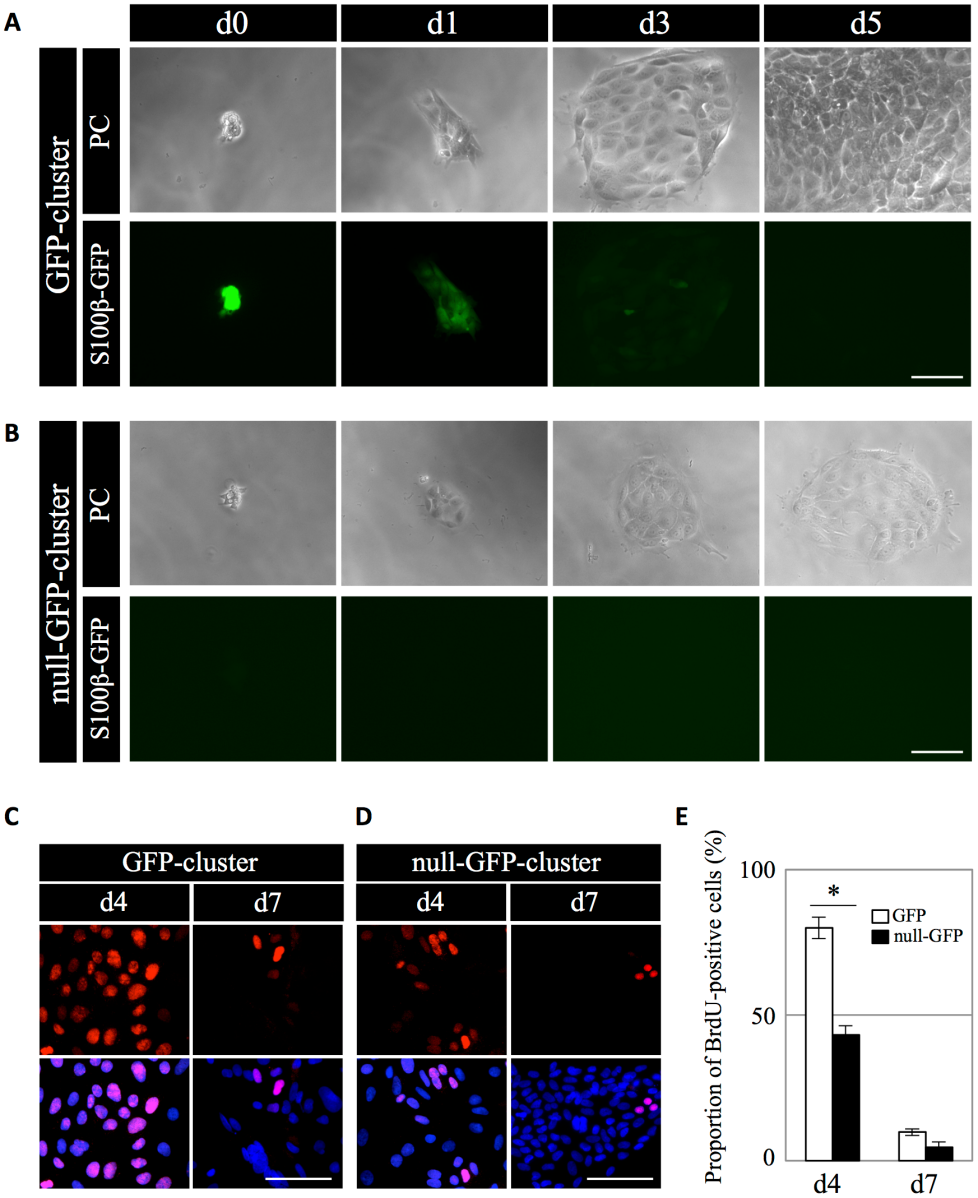
Analysis of the proliferative activity of S100β-positive and -negative PS-clusters upon 2D-cultivation using Matrigel-coated glass slides. (A, B): PS-clusters were isolated from the anterior lobe of adult S100β/GFP-TG rats after enzymatic treatment. Time-lapse images of GFP- (A) and null-GFP-clusters (B) during 2D-cultivation in growth and differentiation-medium (GD-medium) on Matrigel-coated glass slides for 5 days are shown. Phase-contrast (PC) (upper panels) and fluorescence images (lower panels) of each PS-cluster during cultivation. Images were obtained at 0, 1, 3, and 5 days after seeding. Bars: 50 μm. (C, D): Immunostaining for BrdU on GFP- and null-GFP-clusters after 2D-cultivation. Each GFP- (C) and null-GFP-cluster (D) was treated with BrdU for 24 h after days 3 and 6, followed by immunostaining for BrdU. BrdU visualized with Cy3 (*red*) and merged image with nuclear staining by DAPI (*blue*) are shown in upper and lower panels, respectively. Bars: 50 μm. (E): The proportion of BrdU-positive cells in the cells derived from each GFP- and null-GFP-cluster after treatment of BrdU for 24 h from 3 and 6 days-cultivation. White and black bars indicate GFP- and null-GFP-clusters, respectively. The data are presented as the mean ± SE (n = 3) in three independent experiments with triplicate wells. The statistical significance between the groups of GFP- and null-GFP-clusters was determined by Student’s *t*-test. *P < 0.01.

Upon 2D-cultivation in serum-free DMEM/F-12 containing B27, BSA, bFGF, and EGF (GD-medium), GFP-clusters attached on Matrigel and started to proliferate (Fig 1A and 1B). However, further proliferation tended to be inhibited when a single PS-cluster rather than five PS-clusters, was seeded per well (data not shown). Therefore, we performed a subsequent analysis after seeding five PS-clusters per well. S100β-GFP signals in the GFP-clusters decreased and the majority disappeared after 3 day-cultivation (Fig 1A), whereas the signals of null-GFP-clusters did not change throughout cultivation (Fig 1B). In addition, GFP-clusters proliferated more actively than null-GFP-ones (Fig 1A and 1B). During cultivation, using propidium iodide-staining, we confirmed that both subtypes of PS-clusters were composed of living cells (data not shown). To analyze proliferation activity, we treated the cells with BrdU for 24 h after 3 and 6 day-cultivation, followed by immunostaining for BrdU. The results demonstrated that GFP-cluster cell proliferation was active on days 3 to 4 but was almost inactive on days 6 to 7 (Fig 1C and 1D), with a higher rate than that of the null-GFP-clusters (Fig 1E).

**Fig 2:**
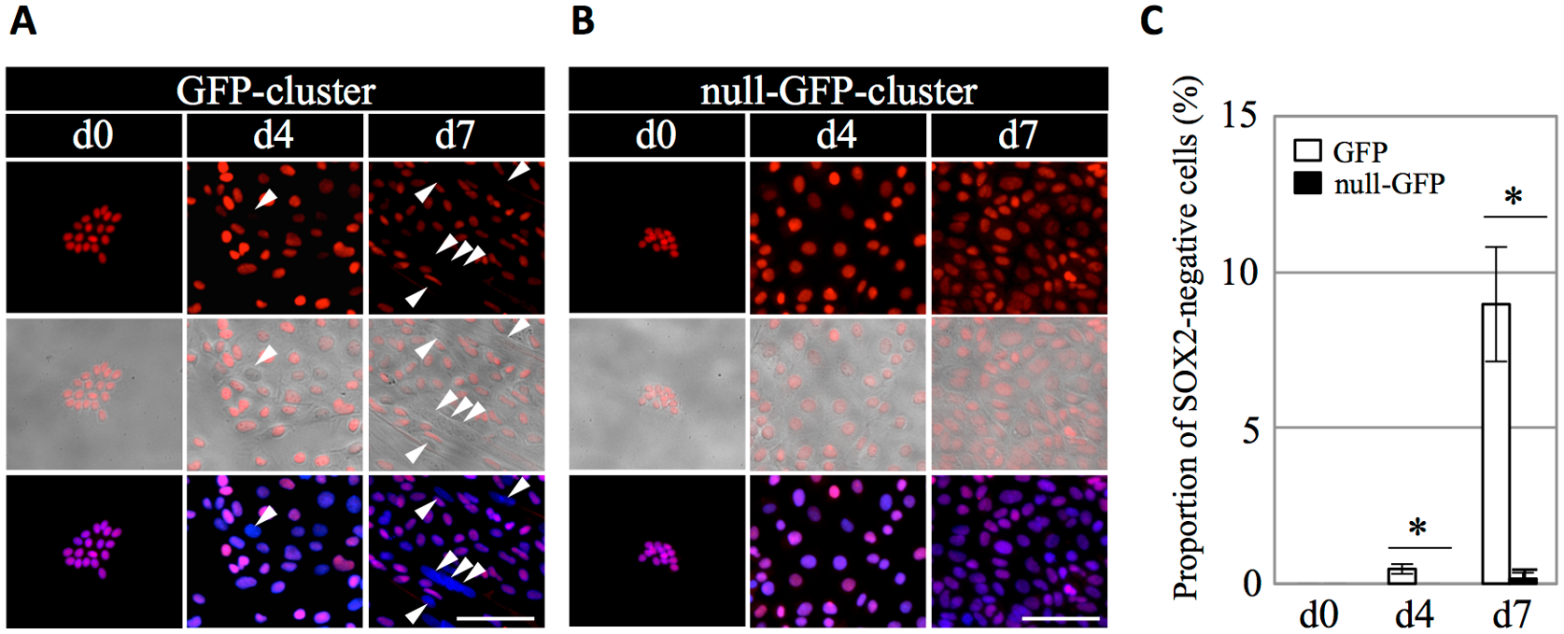
Immunocytochemistry for SOX2 on GFP- and null-GFP-clusters after 2D-cultivation. (A, B): Immunostaining for SOX2 on GFP- (A) and null-GFP-clusters (B) after 2D-cultivation in GD-medium for 0, 4, and 7 days. SOX2 visualized with Cy3 (*red*), merged image with phase-contrast, and nuclear staining by DAPI (*blue*) are shown in upper, middle, and lower panels, respectively. Arrowheads indicate SOX2-negative cells. Bars: 50 μm. (C): Proportion of SOX2-negative cells in the cells derived from each GFP- and null-GFP-cluster after 0, 4, and 7 day-cultivation. White and black bars indicate GFP- and null-GFP-clusters, respectively. The data are presented as the mean ± SE (n = 3) in three independent experiments with triplicate wells. The statistical significance between the groups of GFP- and null-GFP-clusters was determined by Student’s *t*-test. *P < 0.01.

Next, to analyze the undifferentiated state, we performed immunostaining for SOX2 after 0, 4, and 7 day-cultivation (Fig 2). On day 0, immunostaining demonstrated that all cells comprising GFP-clusters, as well as null-GFP-clusters, were SOX2-positive (Fig 2A and 2B). Subsequently, SOX2-negative cells gradually appeared in the cells derived from GFP-clusters on days 4 and 7 (Fig 2A, arrowheads) but not in the cells derived from null-GFP-clusters (Fig 2B). The proportion of SOX2-negative cells in the cells derived from GFP-clusters significantly increased, reaching approximately 9.0% on day 7 (Fig 2C).

### Differentiation capacity of GFP-clusters into pituitary endocrine cell lineages

**Fig 3:**
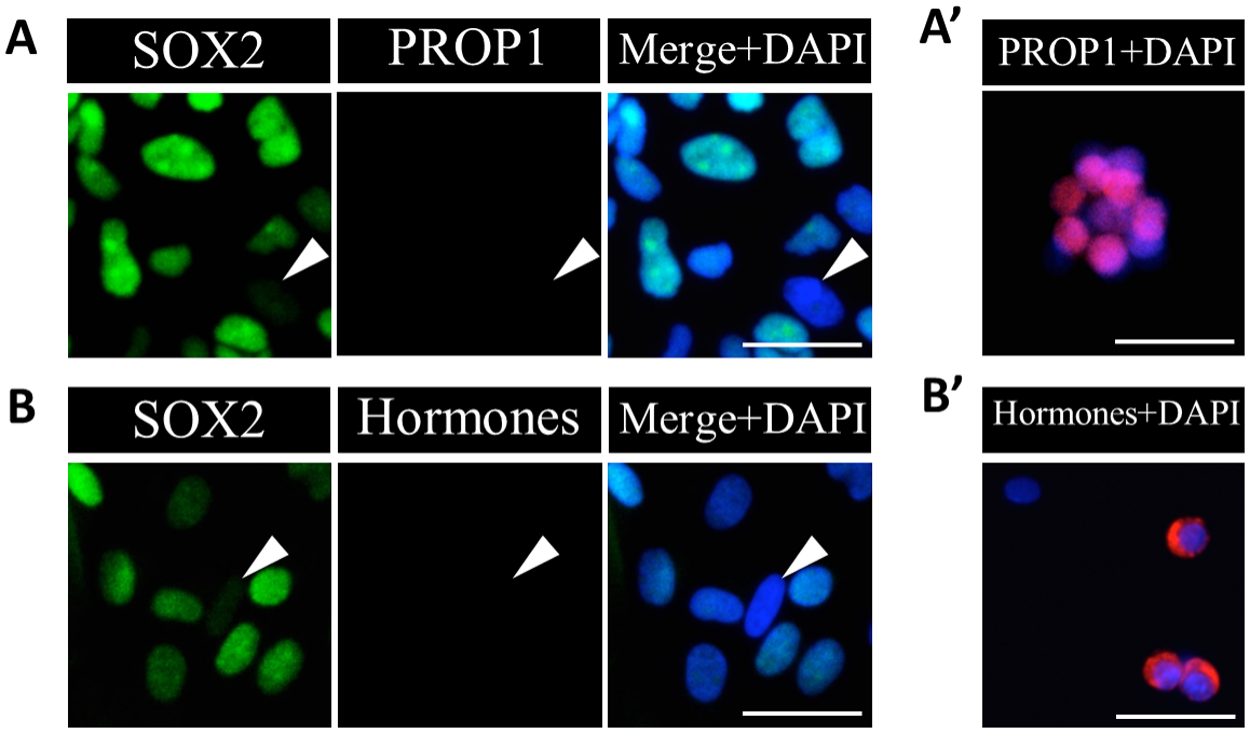
Immunocytochemistry for the pituitary cell lineage markers on GFP-clusters after 2D-cultivation. Immunostaining for PROP1 (A) and pituitary hormones (B) on GFP-clusters after 2D-cultivation in GD-medium for 7 days was performed. SOX2 visualized with Cy5 (*green*), and PROP1 (A) or pituitary hormones with Cy3 (*red*: B), and merged image with nuclear staining by DAPI (*blue*) are shown. Merged images with DAPI and PROP1 (A') or pituitary hormones (B') in the cells before cultivation are also shown. Arrowheads indicate SOX2-negative cells. Bars: 20 μm.

Because GFP-clusters have the capacity to give rise to the cells negative for SOX2, we focused on the differentiation capacity of these clusters. First, to analyze whether GFP-clusters differentiate into pituitary endocrine cells in this 2D-cultivation system, we performed immunostaining for the pituitary specific progenitor cell marker, PROP1, as well as terminally differentiated cell marker hormones (using a cocktail of antibodies against GH, TSHβ, PRL, LHβ, FSHβ, αGSU, and ACTH) after 7 day-cultivation of GFP-clusters under the same conditions described above. Immunostaining for PROP1, which existed in approximately 92% of non-cultured PS-clusters [17], showed that PROP1 had already disappeared from all cells derived from GFP-clusters after cultivation (Fig 3A). Nevertheless, cells derived from GFP-clusters after cultivation were negative for pituitary hormones (Fig 3B). We confirmed that PROP1- or hormones-positive cells were detected in the cells among PS-clusters or dispersed cells before cultivation (Fig 3A′ and 3B′). Moreover, although we tried to induce differentiation on 2D-cultivation using a medium established for 3D-cultivation by Yoshida *et al*. [17] containing BIO (GSK3β-inhibitor), hormone-positive cells could not be detected (data not shown).

### Differentiation capacity of GFP-clusters into non-endocrine cell lineages

**Fig 4:**
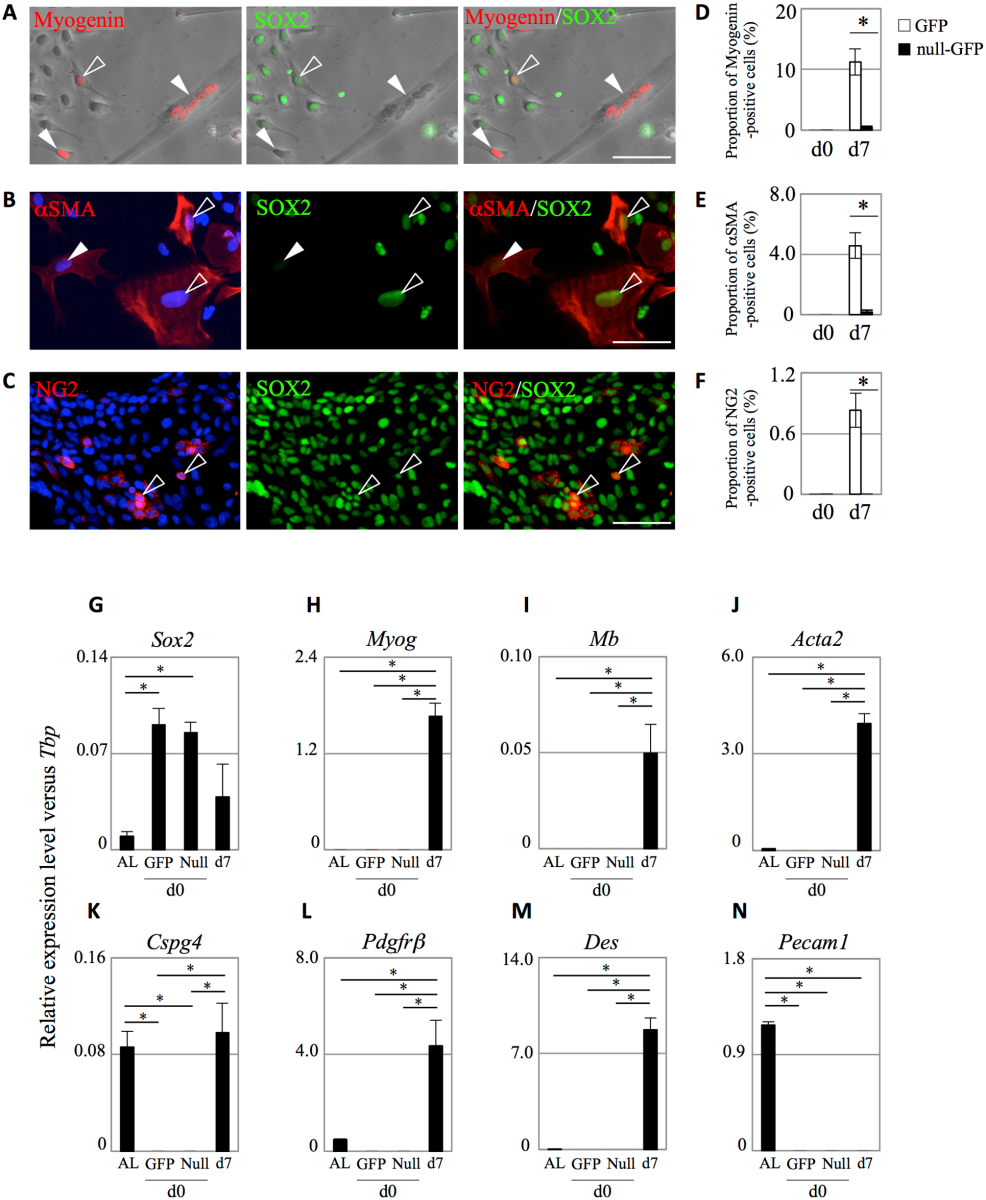
Immunocytochemistry for non-endocrine cell lineage markers on PS-clusters after 2D-cultivation on Matrigel-coated glass slides. (A-C): Immunostaining for SOX2 and non-endocrine cell lineage markers (Myogenin, αSMA, and NG2) was performed on GFP-clusters after 2D-cultivation in GD-medium for 7 days. Each non-endocrine cell lineage factor: Myogenin (A), αSMA (B), and NG2 (C) was visualized with Cy3 (*red*) and SOX2 was visualized with Cy5 (*green*). Merged images with each factor and phase-contrast (A) or nuclear staining by DAPI (*blue*) (B, C) are shown. Open- and closed-arrowheads in the cells positive for each factor indicate typical cells positive and negative for SOX2, respectively. Bars: 50 μm. (D-F): The proportion of Myogenin (D), αSMA (E), or NG2 (F)-positive cells among cells derived from 5 clusters of GFP- or null-GFP-clusters in each well of chamber slides after 0 and 7 day-cultivation. White and black bars indicate GFP- and null-GFP-clusters, respectively. The data are presented as the mean ± SE (n = 3) in three independent experiments with triplicate wells. The statistical significance between the groups of GFP- and null-GFP-clusters was determined by Student’s *t*-test. *P < 0.01. (G-N): Quantitative gene expression analysis of GFP-clusters prior to and following 2D-cultivation in GD-medium for 7 days. Quantitative real-time PCR (qPCR) was performed to estimate mRNA levels of *Sox2* (G), genes related to myogenesis (myogenin: *Myog* in H, and myoglobin: *Mb* in I), alpha-smooth muscle actin (*Acta2* in J), genes related to pericytes (chondroitin sulfate proteoglycan 4: *Cspg4* in K, beta-type platelet-derived growth factor receptor: *Pdgfrβ* in L, and desmin: *Des* in M), and *Pecam1* in N. AL: non-cultured dispersed cells of the anterior lobe, d0-GFP: non-cultured GFP-cluster, d0-Null: non-cultured null-GFP-cluster, and d7: GFP-cluster after 2D-cultivation for 7 days. Each sample was measured in duplicate, and data were calculated using the comparative C_T_ method to estimate copy number relative to that of the TATA box binding protein gene (*Tbp*), which was used as an internal standard. Data are presented as the mean ± SE (n = 3) in three independent experiments. Statistical significance was determined by One-way ANOVA followed by performing Tukey’s test for multiple comparisons. *P < 0.01.

We next investigated differentiation into non-endocrine cell lineages by immunostaining after 7 day-cultivation under the same condition (Fig 4A–4C). We first performed immunostaining for Myogenin, a myoblast marker, as a small population (1.4%) of S100β-positive cells in the rat anterior pituitary shows the capacity to differentiate into skeletal muscle cells [23]. The results demonstrated that Myogenin-positive/SOX2-negative single cells or multinucleated cells forming myotubes emerged from the GFP-clusters (Fig 4A, closed-arrowheads). In addition, Myogenin/SOX2-double positive cells, which represent cells transitioning into differentiation, were also detected (Fig 4A, open-arrowheads). Moreover, we observed that the multinucleated cells forming the myotube started to contract autonomously during cultivation (S1 Movie).

In the anterior lobe, the presence of several non-endocrine cell lineages, mainly cells composed of capillary blood vessels, has been reported. Hence, we also performed immunostaining for the smooth muscle cell marker alpha-smooth muscle actin (αSMA), the pericyte marker neuron-glial antigen 2 (NG2), and the endothelial cell marker platelet endothelial cell adhesion molecule 1 (PECAM1). Immunostaining for αSMA and NG2 demonstrated that the cells positive for each marker were detected among the cells derived from GFP-clusters after cultivation, the majority of which were also positive for SOX2 (Fig 4B and 4C, open-arrowheads). In addition, a few αSMA-positive cells negative for SOX2 were also detected (Fig 4B, closed-arrowheads). In contrast, we could not detect PECAM1-positive cells in this condition (data not shown). We measured the proportion of Myogenin-, αSMA-, and NG2-positive cells among the cells derived from each GFP-cluster after 0 and 7 day-cultivation (Fig 4D–4F). GFP-clusters, which were negative for each marker on day 0, gave rise to cells positive for each marker: Myogenin (11.2%), αSMA (4.6%), and NG2 (0.8%) after 7 day-cultivation (Fig 4D–4F). Null-GFP-clusters showed negative staining for each marker after both 0 and 7 day-cultivation (Fig 4D–4F and S1 Fig).

We also confirmed gene expression levels in GFP-clusters prior to and following cultivation by qPCR (Fig 4G–4N). Expression levels of *Sox2* decreased after cultivation compared to those in non-cultured-GFP-clusters (Fig 4G). Regarding myoblasts, qPCR revealed that *Mb* (encoding Myoglobin, which is expressed in terminally differentiated skeletal muscle) as well as *Myog* (encoding Myogenin) were detected in cultured-GFP-clusters but not in either dispersed cells from the anterior lobe or non-cultured-GFP-clusters (Fig 4H and 4I). Expression levels of *Acta2* (encoding αSMA) (Fig 4J) or the pericyte-related genes *Cspg4* (encoding NG2) (Fig 4K), *Pdgfrβ* (encoding PDGFRβ) (Fig 4L), and *Des* (encoding DESMIN) (Fig 4M) also increased after cultivation compared to those in non-cultured-GFP-clusters. In contrast, *Pecam1* was not detected in GFP-clusters either before or after cultivation (Fig 4N), which corresponding to the results of immunocytostaining (data not shown). These results were confirmed using another internal control gene, *Hprt1* (hypoxanthine phosphoribosyltransferase 1) (data not shown).

**Fig 5:**
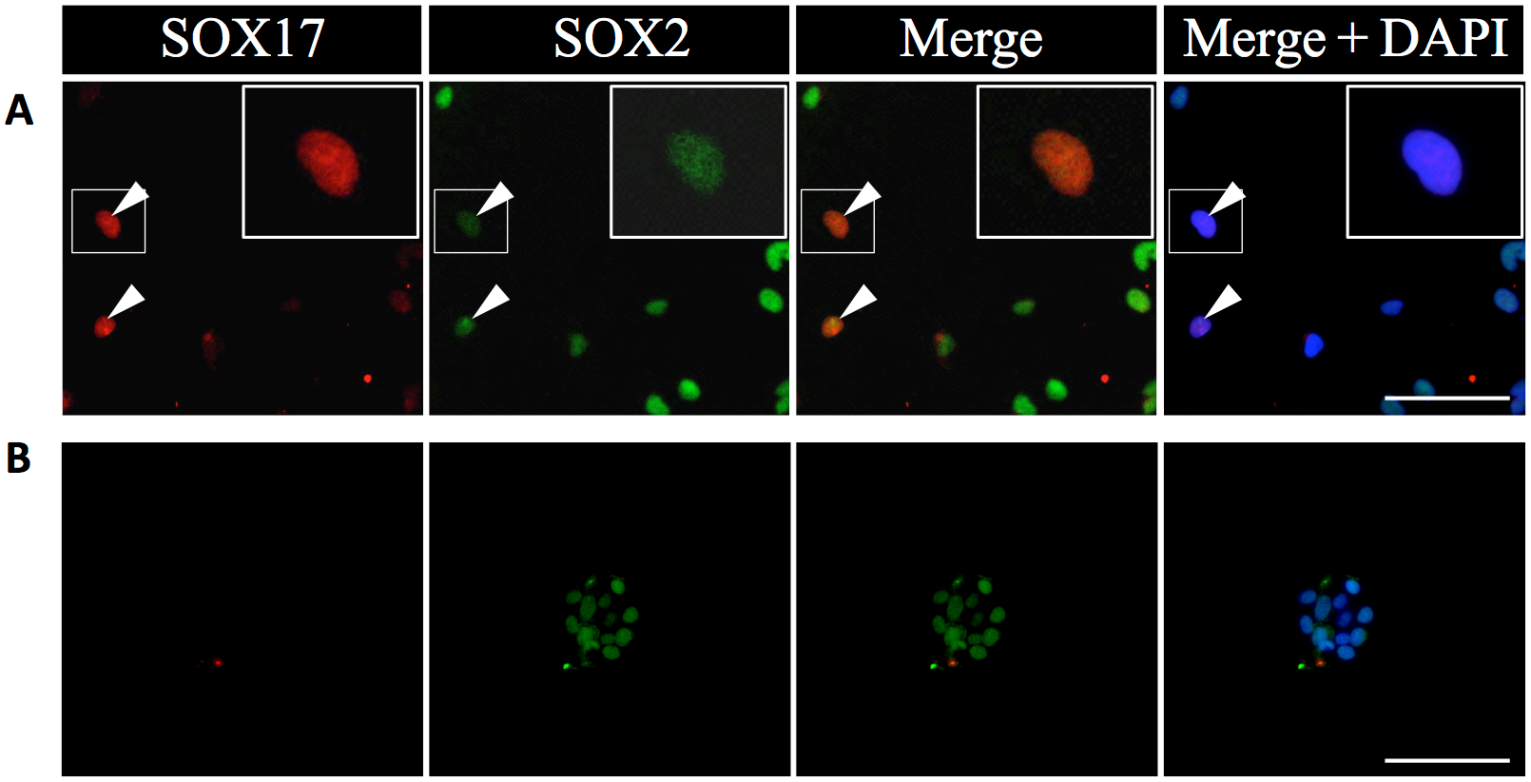
Immunocytochemistry for the primitive endodermal lineage marker, SOX17 on GFP-clusters after 2D-cultivation. Immunostaining for SOX17 and SOX2 on GFP-clusters (A) and null-GFP-clusters (B) after 2D-cultivation in activin-medium for 7 days was performed. SOX17 visualized with Cy3 (*red*), SOX2 with Cy5 (*green*), and merged images without and with nuclear staining by DAPI (*blue*) are shown. Boxed areas are enlarged in each image. Arrowheads indicate SOX17/SOX2-double positive cells. Bars: 50 μm.

The immunopositive cells found following 2D-cultivation as described above were likely to be of mesodermal lineage. We therefore examined whether GFP-clusters had the capacity for differentiation into another lineage using an antibody for SOX17, which is frequently used as a marker for the primitive endoderm lineage. All cells derived from GFP- and null-GFP-clusters were negative for SOX17 after 2D-cultivation using GD-medium for 7 days (data not shown). Notably, treatment with activin-A was shown to differentiate iPS cells into the endodermal lineage cells [24]. Hence, we modified our GD-medium to a medium containing 100 ng/ml activin-A in addition to N2 and BSA, and cultivated GFP-clusters for 7 days. After cultivation, double-immunostaining for SOX17 and SOX2 demonstrated that a few cells derived from GFP-clusters gave rise to cells double-positive for SOX17 and SOX2 (Fig 5A). However, cultivation of null-GFP-clusters in the same conditions did not yield positive cells (Fig 5B) as well as non-cultured GFP- and null-GFP-clusters (S2A and S2B Fig), and dispersed cells of the anterior lobe (S2C Fig).

In summary, GFP-clusters (composed of SOX2-positive cells positive for S100β) but not null-GFP-clusters (composed of SOX2-positive cells negative for S100β) showed the capacity to give rise to cells positive for markers of non-endocrine cells (myoblasts, smooth muscle cells, pericytes, or SOX17-positive cells) depending on 2D-culture conditions.

## Discussion

In the present study, we characterized PS-clusters; particularly, S100β-positive clusters, using a 2D-cultivation system. Collectively, we demonstrated that PS-clusters composed of SOX2-positive cells show different properties with respect to proliferation and differentiation based on the difference in the *S100β*-expression, and that S100β-positive PS-clusters differentiate into non-endocrine cell lineage marker-positive cells depending on the 2D-culture conditions.

Regarding the differentiation capacity of S100β-positive cells, we have reported that a small population of S100β-positive cells in the anterior lobe has the capacity to differentiate into skeletal muscle cells *in vitro* using S100β/GFP-TG rats [23]. However, it is not clear whether pituitary SOX2-positive cells differentiate into this cell type, because S100β-positive cells contain both SOX2-positive and SOX2-negative sub-populations [4]. The present study, therefore, first demonstrated the differentiation capacity of SOX2/S100β-double positive cells in the parenchymal-niche into cells positive for non-endocrine cell lineage markers such as Myogenin, αSMA, NG2, or SOX17 in the 2D-cultivation system, in addition to the endocrine cell lineages assessed by the 3D-cultivation system in our previous report [17]. To maintain the physiological functions of the pituitary, a blood capillary network composed of endothelial cells, smooth muscle cells, and pericytes is required [25]. Although the existence of stem/progenitor cells specific for each endothelial cell, smooth muscle cell, and pericyte is assumed, the present data indicate a possibility that SOX2/S100β-positive cells in the parenchymal-niche contribute to the supply of not only endocrine cells but also cells composing capillary blood vessels in the anterior lobe. However, previous *in vivo* cell tracing studies using *Sox2*^*CreERT2/+*^/*R26*^*YFP/+*^ mice [2] or *Sox9*^*Ires−CreERT2/+*^; *R26*^*YFP/+*^ mice [26] did not discuss the differentiation capacity of pituitary stem/progenitor cells into non-endocrine lineages. Therefore, the issue of whether S100β-positive PS-clusters have the potential to differentiate into non-endocrine cell lineages *in vivo* and the reason why GFP-clusters show different differentiation capacities under 3D- and 2D-cultivation systems remain to be further analyzed.

In the present study, we demonstrated that S100β-positive PS-clusters exhibit the capacity to differentiate into non-endocrine lineages, whereas S100β-negative clusters do not. In comparison, another study used an *in vitro* differentiation assay with a mouse pituisphere-forming system to show that *S100β* gradually starts to be expressed in the process of differentiation [1]. Taken together, these results suggest that S100β-positive PS-clusters might be in more progressive stages toward differentiation than S100β-negative ones, and thus may play important roles in the cell supply system in the adult pituitary. However, most parenchymal-niches as well as S100β-positive cells start to appear during the early postnatal period in rats rather than in the embryonic periods, when stem/progenitor cells actively differentiate, suggesting that mechanisms in the differentiation process may differ between embryonic and postnatal development. Conversely, S100β-negative PS-clusters sustained *Sox2*-expression even after induction of differentiation in this study, showing low proliferation and differentiation activities. Although we could not elucidate whether these clusters had the capacity to express *S100β*, it remains a possibility that the S100β-negative PS-cluster population might be in a quiescent state in the adult rat pituitary.

Another important issue is the heterogeneity of the S100β-positive PS-cluster. Our previous [17] and present study showed that a limited number of S100β-positive PS-clusters and/or particular cells composed an S100β-positive PS-cluster that exhibited the capacity for differentiation into endocrine or/and non-endocrine cells. These data show that S100β-positive PS-clusters harbor heterogeneity, and suggest that some are directed toward differentiation. Moreover, these results raise two hypotheses, that each S100β-positive PS-cluster has (1) multipotent progenitor cells, or (2) unipotent progenitor cells. This issue remains to be elucidated as we could not develop a common differentiation condition for both endocrine and non-endocrine cells from a single PS-cluster. In addition, in the present study, a differentiation condition for the non-endocrine cell lineage induced S100β-positive PS-clusters to differentiate into three types of mesodermal marker-positive cells. However, we could not conclude whether a single S100β-positive PS-cluster exhibited multiple differentiation capacities for these three types of cells, as the differentiation of a PS-cluster was detected only in the intermingled PS-clusters cultured in the same well. Differentiation of individually cultured PS-clusters has not yet been detected suggesting the importance of cell-to-cell communication through paracrine factors and/or direct cell-to-cell interactions to stimulate PS-clusters. Collectively, to clarify the issue of whether S100β-positive PS-clusters harbor multipotent or unipotent differentiation capacity, the development of more effective differentiating conditions to analyze single PS-clusters is necessary.

In summary, we have demonstrated that S100β-positive PS-clusters exhibit high proliferation activity and differentiate into cells positive for non-endocrine cell lineage markers in addition to endocrine cells depending on the culture conditions, whereas S100β-negative PS-clusters show low proliferation and differentiation activity. Therefore, further analysis of the heterogeneity of SOX2-positive cells might provide insight into the processes of pituitary development throughout the lifespan of an organism.

## Supporting information

S1 Fig Immunocytochemistry for non-endocrine cell lineage markers on null-GFP-clusters after 2D-cultivation on Matrigel-coated glass slides. (A-C): Immunostaining for SOX2 and non-endocrine cell lineage markers (Myogenin, αSMA, and NG2) was performed on null-GFP-clusters after 2D-cultivation in GD-medium for 7 days. Each non-endocrine cell lineage factor: Myogenin (A), αSMA (B), and NG2 (C) was visualized with Cy3 (*red*) and SOX2 was visualized with Cy5 (*green*). Merged images with each factor and phase-contrast (A) or nuclear staining by DAPI (*blue*) (B, C) are shown. Bars: 50 μm.
(TIFF)

S2 Fig Immunocytochemistry for SOX17 on GFP-clusters before 2D-cultivation and dispersed cells of the anterior lobe. Immunostaining for SOX17 and SOX2 on GFP- (A) and null-GFP-clusters (B), and dispersed cells of the anterior lobe (C) before 2D-cultivation was performed. SOX17 visualized with Cy3 (*red*), SOX2 with Cy5 (*green*), and merged images without and with nuclear staining by DAPI (*blue*) are shown. Bars: 50 μm.
(TIFF)

S1 Movie Autonomously contracting myotube of cultured-GFP-clusters. Movie showing autonomously contracting myotube derived from GFP-clusters after 2D-cultivation in GD-medium for 7 days. Arrowheads indicate typical contracting cells. Bars: 50 μm.
(PPTX)

S1 Table List of primary antibodies. (TIF)

S2 Table List of primer sets for real time PCR. (TIF)
