# Supplementary figures and images for: Differentiation capacities of PS-clusters, adult pituitary stem/progenitor cell clusters located in the parenchymal-niche, of the rat anterior lobe
Source: PLoS One. 2018 Apr 23;13(4):e0196029. doi: 10.1371/journal.pone.0196029 (PMC5912746; doi:10.1371/journal.pone.0196029)

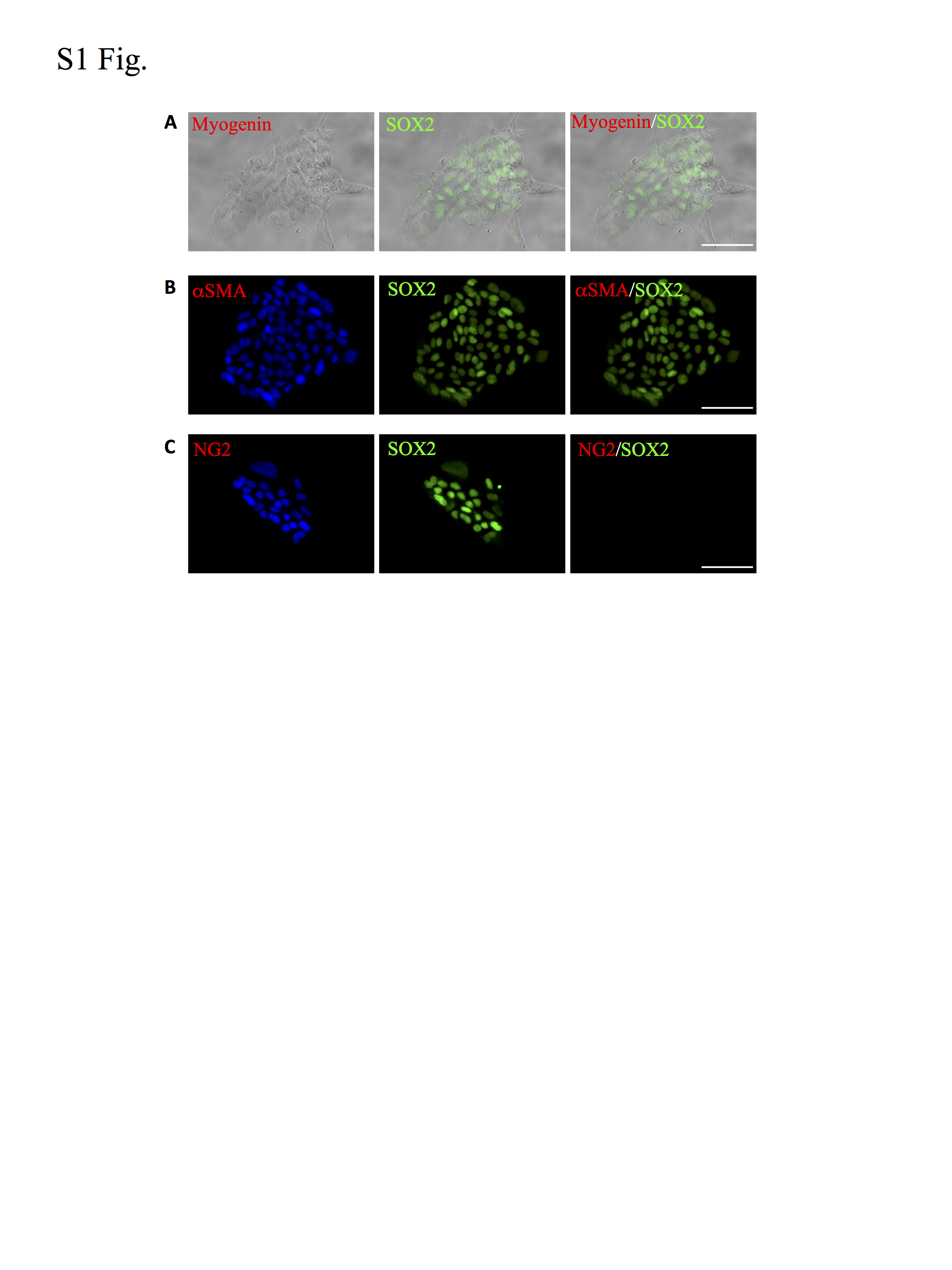

Supplement: S1 Fig — (A-C): Immunostaining for SOX2 and non-endocrine cell lineage markers (Myogenin, αSMA, and NG2) was performed on null-GFP-clusters after 2D-cultivation in GD-medium for 7 days. Each non-endocrine cell lineage factor: Myogenin (A), αSMA (B), and NG2 (C) was visualized with Cy3 (red) and SOX2 was visualized with Cy5 (green). Merged images with each factor and phase-contrast (A) or nuclear staining by DAPI (blue) (B, C) are shown. Bars: 50 μm. (TIFF) [file pone.0196029.s001.tiff]

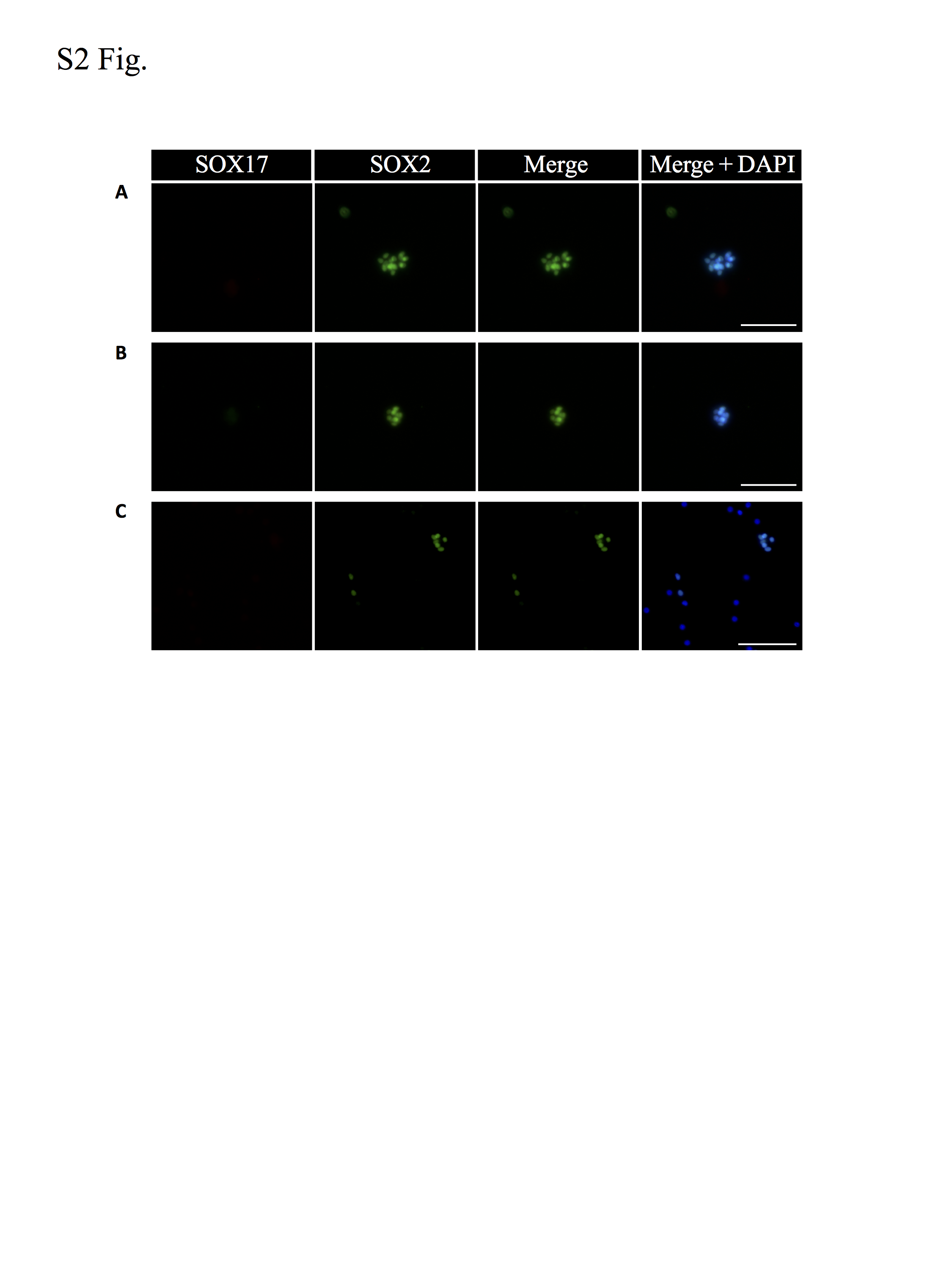

Supplement: S2 Fig — Immunostaining for SOX17 and SOX2 on GFP- (A) and null-GFP-clusters (B), and dispersed cells of the anterior lobe (C) before 2D-cultivation was performed. SOX17 visualized with Cy3 (red), SOX2 with Cy5 (green), and merged images without and with nuclear staining by DAPI (blue) are shown. Bars: 50 μm. (TIFF) [file pone.0196029.s002.tiff]

## Slide 1
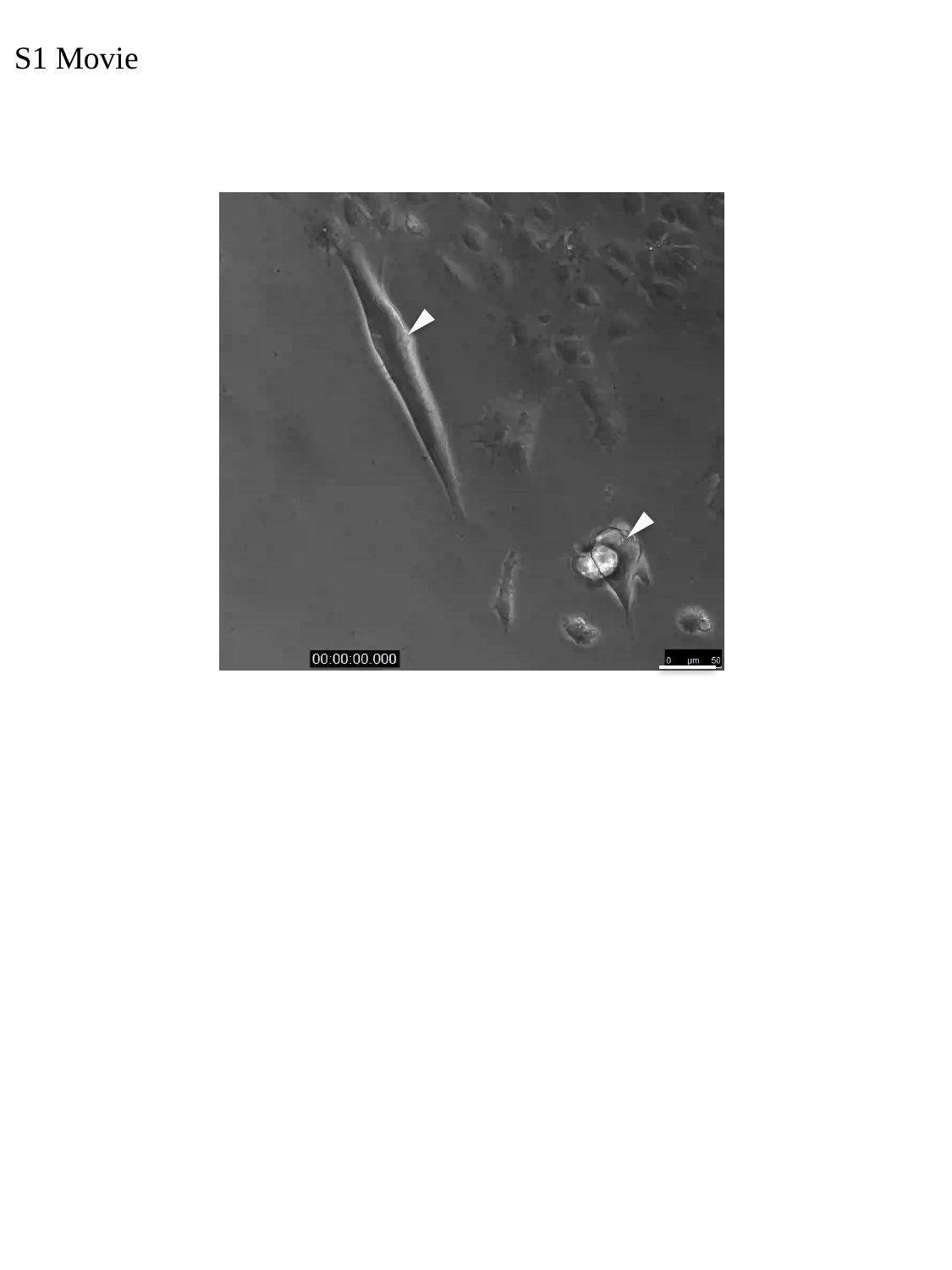

S1 Movie

Supplement: S1 Movie — Movie showing autonomously contracting myotube derived from GFP-clusters after 2D-cultivation in GD-medium for 7 days. Arrowheads indicate typical contracting cells. Bars: 50 μm. (PPTX) [file pone.0196029.s003.pptx]

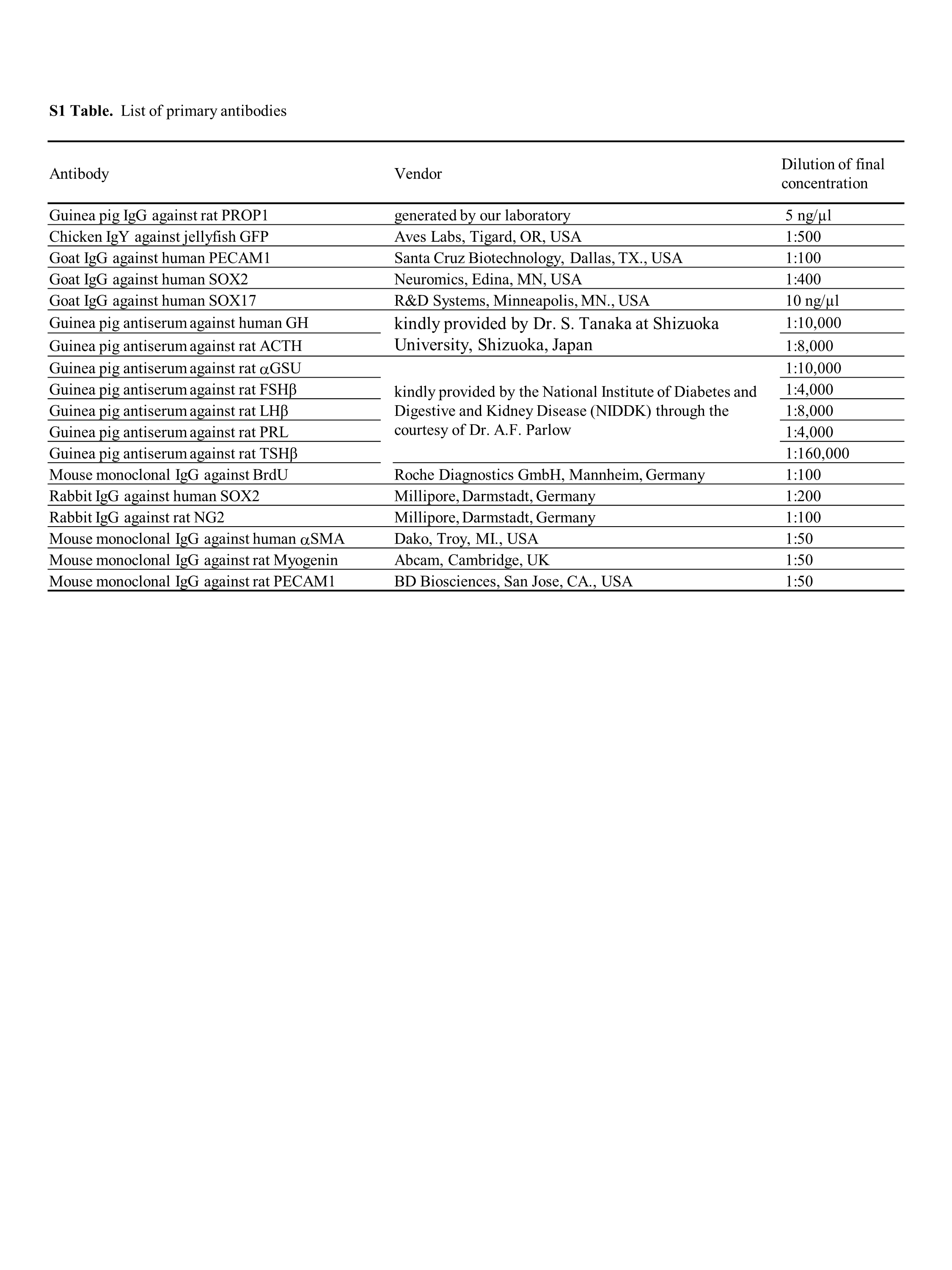

Supplement: S1 Table — (TIF) [file pone.0196029.s004.TIF]

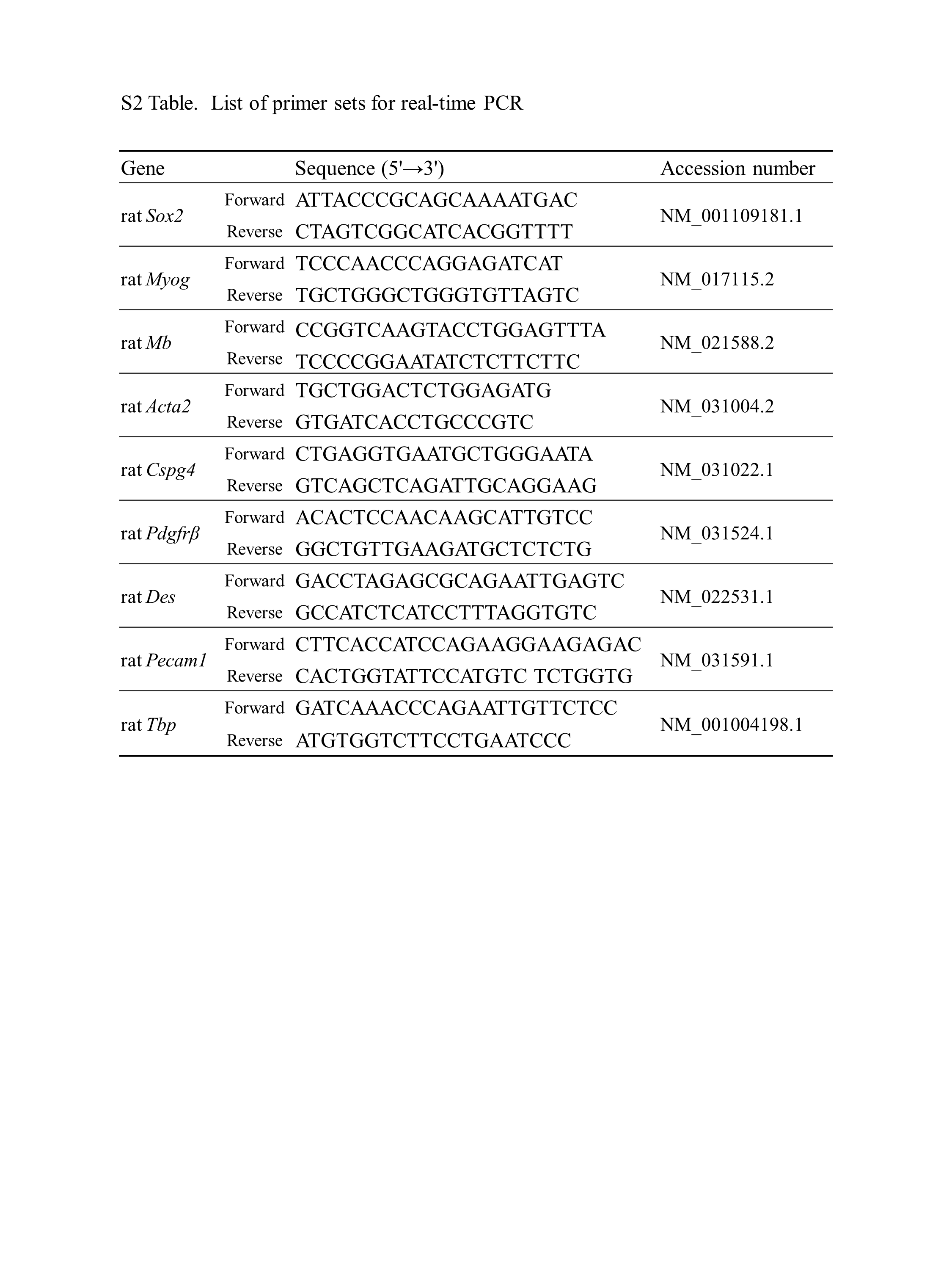

Supplement: S2 Table — (TIF) [file pone.0196029.s005.TIF]
